# Long-Term Cognitive, Functional, and Patient-Reported Outcomes in Patients With Anti-NMDAR Encephalitis

**DOI:** 10.1212/WNL.0000000000210109

**Published:** 2024-11-20

**Authors:** Juliette Brenner, Cinthia J. Ruhe, Ilse Kulderij, Anna E.M. Bastiaansen, Yvette S. Crijnen, Chelsey N. Kret, Julia C.P. Verkoelen, Anke A.G. Tolido, Brigit Thomassen, Laura P. Kersten, Marienke A.A.M. de Bruijn, Sammy H.C. Olijslagers, Melissa R. Mandarakas, Jeroen Kerstens, Robin W. van Steenhoven, Juna M. de Vries, Sharon Veenbergen, Marco W.J. Schreurs, Rinze F. Neuteboom, Peter A.E. Sillevis Smitt, Esther van den Berg, Maarten J. Titulaer

**Affiliations:** From the Department of Neurology (J.B., C.J.R., I.K., A.E.M.B., Y.S.C., C.N.K., J.C.P.V., A.A.G.T., B.T., L.P.K., M.A.A.M.d.B., M.R.M., J.K., R.W.v.S., J.M.d.V., R.F.N., P.A.E.S.S., M.J.T.), Erasmus University Medical Center, Rotterdam; Department of Neurology (S.H.C.O.), Amsterdam University Medical Center; Department of Immunology (S.V.), Erasmus University Medical Center, Rotterdam; Laboratory of Medical Microbiology and Immunology Microvida (M.W.J.S.), Tilburg; and Department of Neurology & Alzheimer Center (E.v.d.B.), Erasmus University Medical Center, Rotterdam, the Netherlands.

## Abstract

**Background and Objectives:**

Anti-NMDA receptor (anti-NMDAR) encephalitis generally manifests in young adults. Although 80%–90% returns to independence, the majority experience persistent cognitive and psychosocial difficulties. Studies have demonstrated that cognitive recovery may continue for years; the temporal trajectory is largely unknown, as are factors influencing cognitive/psychosocial recovery. Objectives were to (1) describe the cognitive recovery trajectory, (2) assess self-reported outcomes, (3) identify factors relating to outcome, and (4) explore the relation between cognitive and self-reported outcomes, and participation.

**Methods:**

We performed a large-scale cross-sectional and prospective cohort study. We addressed our nationwide cohort, provided they were (1) older than 16 years, (2) independent preillness, and (3) able to perform cognitive tests and/or self-report. Patients completed Patient-Reported Outcome Measures and neuropsychological assessments (memory, language, perception and construction, and attention and executive functions), and functional outcomes were established (modified Rankin Scale [mRS] score and return-to-work/-education). Outcomes were compared with references and between groups based on clinical characteristics and functional outcomes (*T*-tests for normalized data and nonparametric tests for patient-reported data). Recovery was visualized by plotting outcomes against time-of-assessment.

**Results:**

We included 92 patients (age 29 ± 2 years; 77% female). Cognitive scores improved with time-of-assessment, up to 36 months after diagnosis (*R* = 0.35, *p* = 0.022), with the most enhanced improvement in the first 6 months. This result could be reproduced in prospective patients (n = 12). Beyond 36 months (n = 44), 34% of patients had a persistent impairment (*z*-score <−1.5 SD) and 65% scored below-average (<−1 SD) in 1 or more cognitive domains, despite a “favorable” outcome measured by mRS (≤2) in the majority (91%). Most affected were memory (mean −0.67 ± 0.89 SD, *p* = 0.25) and language (−0.75 ± 1.06 SD, *p* = 0.23). Self-reported complaints remained in emotional well-being (mean 72 ± 25 SD vs norm 82 ± 33 SD, *p* < 0.001), social functioning (73 ± 26 SD vs 84 ± 22 SD, *p* < 0.001), energy levels (57 ± 19 SD vs 69 ± 19 SD, *p* < 0.001), and quality of life (0.85 ± 0.14 SD vs 0.93 ± 0.11 SD, *p* < 0.001). Many patients did not resume school/work (30%) or needed adjustments (18%). Resuming school/work related to processing speed (−0.14 ± 0.78 SD vs −0.84 ± 1.05 SD, *p* = 0.039) and well-being (EuroQol 5 Dimensions 5 Levels median 0.90 vs 0.81, *p* = 0.016).

**Discussion:**

Recovery from anti-NMDAR encephalitis may continue for 3 years, with risk of persisting cognitive deficits, notably in memory and language, and sequelae in social functioning, energy levels, and well-being. The frequently applied outcome measure mRS does not fully capture outcomes. Almost half of patients struggled resuming school/work, associated with cognitive deficits and well-being.

## Introduction

Anti-NMDA receptor (anti-NMDAR) encephalitis generally manifests in young adults between the ages of 15 and 30.^1^ Autologous antibodies target the NMDARs in their brain. These excitatory receptors are diffusely present in the brain and involved in many processes, such as one of the foremost memory formations. The interaction of antibodies with the receptors, and cerebral inflammation, cause subacute cognitive disturbances and other neurologic and neuropsychiatric symptoms.^2,3^ The outcome of this often monophasic disease is believed to be favorable in most patients (≥80%), defined as independence in daily activities in 1 year^3^ or 2 years^4^ after diagnosis—or a score of ≤2 on the modified Rankin Scale (mRS). These numbers, however, are based on clinical outcome assessments not ideal for capturing the long-term effects of encephalitis.^5^ By contrast, cognitive impairments frequently (∼80%) persist and pose significant challenges to the relatively young patients' daily lives.^6,7^

As opposed to the often rapid recovery of neurologic symptoms of anti-NMDAR encephalitis such as seizures and movement disorders, the recovery of cognitive deficits is believed to take months to years.^3,6,8^ Studies have revealed deficits across all cognitive domains, years after the acute phase. Deficits were identified in verbal and visual memory,^6,9-18^ executive function,^10-13,19-22^ attention and working memory,^22-24^ language,^16,17,19^ and visuospatial function.^24^ Particularly, deficits in the memory and executive function may persist long after diagnosis.^6,25^ Residual cognitive deficits have also been self-reported by patients, as having poor memory,^23,26,27^ difficulty concentrating,^23,26^ or increased fatigability.^23,26,28^ In addition, patients have reported consequences for their quality of life and emotional well-being,^23,29,30^ not always correlating with objective cognitive symptoms.^16^ Self-reported difficulties with attention/concentration have been linked to 25%–33% of patients with anti-NMDAR encephalitis not returning to their previous work or education.^16,31-33^

Although studies have investigated cognitive recovery within the first year after diagnosis^34^ and beyond,^6^ a large-scale assessment of cognitive recovery over time, as well as the link to patient-reported outcomes is not available. The relation between objective cognitive sequelae of anti-NMDAR encephalitis and the probability to return to work/education has not previously been investigated.

Identifying factors contributing to persistent cognitive deficits and promoting cognitive recovery is paramount in preserving the patients' potential to reintegrate into society. Factors that have previously been related to outcome include patient factors such as age^1,35^; disease factors such as treatment delay, maximum disease severity (including intensive care unit [ICU] admission), and lack of response to first-line therapy^4,36^; and serologic, radiologic, and electrophysiologic biomarkers. Leukocyte count in the CSF is an accessible immunologic biomarker,^36^ hippocampal involvement is an evident radiologic biomarker,^11,36-38^ and electrophysiologic markers include an abnormal posterior rhythm.^39,40^ Most prognostic studies used the mRS as an outcome measure, instead of detailed neuropsychological assessments.^41^ Only a decreased consciousness, delayed treatment, and lack of response to treatment have been related to poorer cognitive outcome.^11,34,42^ In patients with a preceding herpes simplex virus encephalitis (HSVE) to the anti-NMDAR encephalitis, notable impairments in memory test performance were observed, alongside occasional inferior language performance.^43^

In this nationwide cohort study, we describe the full scope of long-term outcomes of anti-NMDAR encephalitis. The objectives are to (1) describe the trajectory of cognitive recovery, (2) assess long-term patient-reported outcomes, (3) identify clinical factors relating to long-term outcome, and (4) explore the relation between long-term cognitive outcomes and self-reported physical and emotional well-being, daily functioning, participation in society, and quality of life.

## Methods

### Participants

**Figure 1:**
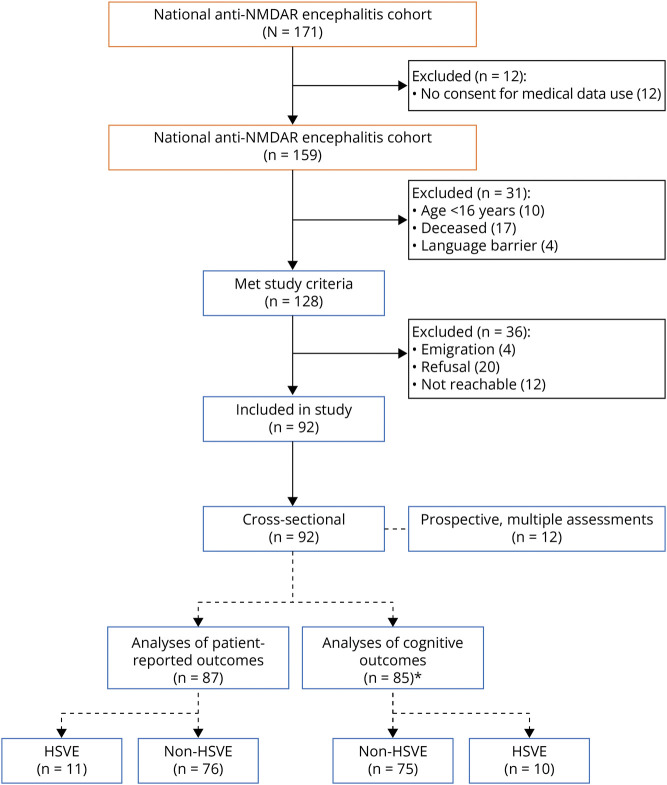
Patient Selection At the end of the inclusion period (July 2023), 171 patients had been diagnosed with an anti-NMDAR encephalitis in the Netherlands of whom 159 consented to the use of their medical information. From 127 eligible patients, 92 patients were included in the study of whom 85 completed the cognitive assessment (*1 patient excluded from the analysis because of dependence on a speech-device) and 87 completed the PROMs. Twelve patients were within 2 years from diagnosis at the moment of inclusion or were diagnosed during the inclusion period, and had a minimum of 2 assessments. These patients were included in the prospective analyses. The first moment of data collection is also included in the cross-sectional data set. Eleven patients had a preceding HSVE, 10 of these patients had a cognitive assessment; these results were analyzed separately. HSVE = herpes simplex virus encephalitis; PROM = patient-reported outcome measure.

The Erasmus University Medical Center (Erasmus MC) is accredited as European Reference Network Site (ERN-RITA) for autoimmune encephalitis, meaning that all samples from patients suspected for autoimmune encephalitis are tested for antibodies at the Erasmus MC, providing nationwide coverage of patients diagnosed with autoimmune encephalitis, including anti-NMDAR encephalitis. All patients diagnosed in the Netherlands before July 2023, according to the diagnostic criteria for anti-NMDAR encephalitis,^44^ were contacted by the authors to participate in this study, if they were (1) at least 16 years old at the time of the study, (2) functionally independent (mRS ≤2) before the encephalitis, and (3) able to participate in cognitive tests and/or self-report. The ability to participate was based on (1) the level of consciousness/responsiveness (Glasgow Coma Scale score ≥12) and (2) fluency in the Dutch language (Figure 1). All participants were seen by the authors, in the context of the study. If participants were unable to visit the Erasmus MC, the clinical evaluation and cognitive assessment were performed by the authors at the local clinic or participants' home, to minimize selection bias.

For the prospective part of the study, patients who were within 2 years after diagnosis were contacted for multiple assessments, with at least 6 months in-between assessments to minimize a potential learning effect. The first assessments of the prospectively included patients were also included in the cross-sectional analyses (Figure 1).

Previous research and our own current data (eTable 1) suggest that patients with a preceding HSVE may have a distinct cognitive recovery, and distinct clinical parameters, such as more frequent radiologic abnormalities and elevated biomarker levels.^45-47^ A preceding HSVE was not an exclusion criterion for participation in the study; however, patients with a preceding HSVE were excluded from the analysis of cognitive and self-reported recovery over time and prognostic factors, to uphold the integrity of the analysis and reduce potential sources of bias. We did compare the outcomes of these patients with the outcomes of anti-NMDAR encephalitis patients without a preceding HSVE.

### Standard Protocol Approvals, Registrations, and Patient Consents

Written informed consent was obtained from all participants. The study protocol was approved by the institutional review board of the Erasmus MC (NL77821.078.21).

### Clinical Data

Information on the disease episode (patient characteristics, symptoms, ancillary testing, treatment and recovery; Supplementary data dictionary) was obtained by the authors from medical records retrieved from the referring hospitals. If patients had experienced a relapse in the 36 months before cognitive testing, data from the most recent episode were collected. In all other cases, clinical data from the first or only disease episode were collected. Radiologic and serologic findings were considered only if obtained within 90 days of disease onset, to ensure an accurate relation to disease activity.

Functional status at the moment of assessment was determined on the mRS.

### Patient-Reported Outcome Measures

We selected 7 patient-reported outcome measures (PROMs) identified in a recent systematic review about outcome measurement in encephalitis.^5^ The PROMs were the 36-Item Short-Form Health Survey II (SF-36-II),^e1^ World Health Organization Disability Assessment Schedule (WHO-DAS-II),^e2^ Fatigue Severity Scale (FSS),^e3^ Beck Depression Inventory (BDI),^e4^ Hospital Anxiety and Depression Scale (HADS),^e5^ 5-Level 5-Dimension EuroQoL (EQ-5D-5L),^e6^ and World Health Organization-5 Well-being Index (WHO-5).^e7^ For the analysis, we classified them into the constructs “Physical functioning” (FSS, physical domains SF-36-II and WHO-DAS-II), “Daily activities and participation” (WHO-DAS-II), “Emotional well-being” (BDI, HADS, emotional well-being domains SF-36-II), and “Quality of Life” (EQ-5D-5L and WHO-5). Participants completed the PROMs online through the Castor Clinical Trial Platform^e8^ preceding the clinical assessment (median time between self-reported and clinical assessment 5 days, interquartile range 0–24), with a prespecified maximum deviation time to ensure comparability.

### Neuropsychological Assessment

All participants were invited for a cognitive test battery, covering the 4 major cognitive domains (Memory, Language, Perception and Construction, and Attention and Executive functions), constructed from 7 subdomains (eFigure 1). We assessed (1) verbal memory with the Auditory Verbal Learning Test (immediate recall 1–5, delayed recall), (2) visuospatial memory with the delayed recall component of the Rey-Osterrieth Complex Figure Test (ROCFT; 36-point scoring), (3) language with the Boston Naming Test, (4) perception and construction with the Judgement of Line Orientation and copy component of the ROCFT, (5) executive functions with the Brixton Spatial Anticipation Test (number or errors), semantic and phonemic fluency tests and Trail Making Tests (TMT) parts A and B, (6) processing speed with the TMT B/A ratio and the Letter Digit Substitution Test, and attention and working memory with the Wechsler Adult Intelligence Scale-IV Digit Span. All tests were validated in the Netherlands, administered in the official Dutch versions by the authors who had been trained in neuropsychological testing, and had normative data available.^e9^ The average time to complete the battery was 60–120 minutes.

### Statistical Analysis

Scores on the cognitive test components were standardized based on age, sex, and education level, by comparison with normative data of more than 150.000 participants in Advanced Neuropsychological Diagnostics Infrastructure-Norms.^e10^ Scores for the cognitive (sub)domains were calculated by averaging *z*-scores for individual tests into the subdomain scores and subsequently averaging these into the domain scores (eFigure 1). A *z*-score of more than 1.5 SD below the norm-average was considered an impairment, a score of −1 to −1.5 SD “below-average.” If participants were unable to complete a test (n = 2), domain scores were calculated with the remaining test scores. If patients were unable to complete all tests of a domain (n = 3), the scores were set at −3 SD to make maximum use of the information; not being able to perform a cognitive test (in a responsive participant) does provide information on the limited cognitive abilities. The cognitive data of 1 participant were excluded because she relied on a speech-generator, causing her to respond slower in time-sensitive tasks, not reflecting her cognitive abilities.

To identify normative data for the PROMs, we have searched articles describing self-reported outcomes of healthy participants with similar demographics as the included patients in our study (eTable 2). The patient-reported outcomes in our study were compared with the reported means from the reference articles with *T*-tests.

Clinical variables were tested for normality with the Shapiro-Wilk test. Skewed variables were transformed by (1) winsorizing at the 95th-percentile (*diagnostic delay*) and (2) taking the logarithm (*diagnostic and treatment delay*, *CSF leukocytes*, *antibody titer*) or square-root (*age*, *follow-up duration*, and *time to resume work/school*). Reported values are geometric descriptive statistics.

All statistical analyses were performed in R statistical software (version 4.3.2; R Core Team, Vienna, Austria, 2023).^e11^ *T*-tests were performed to compare standardized cognitive data between groups. Analyses on patient-reported outcomes were nonparametric. In the exploratory analyses of prognostic factors for outcome, we have restrained the number of statistical tests by looking at the cognitive domains and not at the individual test level. We controlled for the increased risk of type I errors due to multiple testing, by applying the Holm-Bonferroni method^e12^ to adjust *p*-values per hypothesis,^e13^ as indicated for the individual analyses in the table captions. In addition to statistical significance, we considered a Cohen *d* effect size of >0.5 or a difference in group means exceeding a minimal (clinically) important difference of half the SD of the reference population as a clinically relevant difference.^e14^ Recovery trajectories were explored by visualizing the relation of (cross-sectional and prospectively measured) cognitive and patient-reported outcomes to passed time after diagnosis, fitting local polynomial regression lines and simplifying by fitting linear regression lines with splines, placing knots at the evident time points based on the local polynomials. Analyses directed at identifying prognostic factors were limited to long-term outcomes, defined as outcomes *after* the identified recovery period (36 months), to secure that relations are not based on chance due to the timing of an outcome measurement in the recovery process, rather than the investigated prognostic factor.

### Data Availability

Any data not published within this article are available at Erasmus MC. Patient-related data will be shared on request from any qualified investigator, maintaining anonymization of the individual patients.

## Results

We identified 159 patients with anti-NMDAR encephalitis of whom 92 were enrolled in this study (mean age 29 ± 2 years; 77% female). Eighty-five performed the cognitive tests, 87 the PROMs, and 12 were assessed prospectively for several times (Figure 1). The included patients were largely comparable with the patients in the complete nationwide cohort not included (Table 1), although less severely affected (32% required ICU-admission vs 58%, *p* < 0.001) and less frequently related to a tumor (17% vs 34%, *p* = 0.014). The most severely affected patients in the nationwide cohort were often young children not able to perform the tests (n = 10), or deceased (n = 17, of whom 65% were admitted to the ICU).

**Table 1:** Baseline Characteristics of the Included Patients vs Characteristics of the Nationwide Anti-NMDAR Encephalitis Cohort

	Inclusion in the study		Overall (n = 159)	*p* Value
	Yes (n = 92)	No (n = 67)		
Baseline characteristics				
Sex, female, n/N (%)	71/92 (77)	46/67 (69)	117/159 (74)	0.23^a^
Age at disease onset, y, GM (GSD; min-max)	29 (2; 7–73)	30 (5; 1–87)	29 (3; 1–87)	0.59^b^
Tumor, n/N (%)	16/92 (17)	23/67 (34)	39/159 (25)	0.014^a^
Prior HSVE, n/N (%)	11/92 (12)	9/67 (13)	20/159 (13)	0.78^a^
Clinical symptoms, n/N (%)				
Behavioral changes, n/N (%)	86/92 (93)	62/67 (93)	148/159 (93)	>0.99^a^
Cognitive disturbances, n/N (%)	87/92 (95)	58/67 (87)	145/159 (91)	0.079^a^
Language disorder, n/N (%)	63/92 (68)	45/67 (67)	108/159 (68)	0.86^a^
Seizures, n/N (%)	56/92 (61)	45/67 (67)	101/159 (64)	0.42^a^
Movement disorder, n/N (%)	45/92 (49)	45/67 (67)	90/159 (57)	0.022^a^
Sleep disorder, n/N (%)	42/92 (46)	27/63 (43)	69/155 (45)	0.73^a^
Altered consciousness, n/N (%)	24/92 (26)	35/67 (52)	59/159 (37)	<0.001^a^
Autonomic dysfunction, n/N (%)	35/92 (38)	35/66 (53)	70/158 (44)	0.061^a^
ICU admission, n/N (%)	29/92 (32)	39/67 (58)	68/159 (43)	<0.001^a^
Ancillary testing				
MRI abnormalities, n/N (%)	35/83 (42)	19/62 (31)	54/145 (37)	0.16^a^
White matter abnormalities, n/N (%)	1/83 (1.2)	0/62 (0)	1/145 (0.7)	>0.99^c^
Limbic abnormalities, n/N (%)	22/83 (27)	9/62 (15)	31/145 (21)	0.081^a^
Hyperintense thalamic, n/N (%)	7/83 (8.4)	3/62 (4.8)	10/145 (6.9)	0.52^c^
Other abnormalities, n/N (%)	7/83 (8.4)	7/62 (11)	14/145 (9.7)	0.56^a^
EEG abnormalities, n/N (%)	64/80 (80)	47/51 (92)	111/131 (85)	0.059^a^
Encephalopathic, n/N (%)	46/80 (58)	36/50 (72)	82/130 (63)	0.10^a^
Epileptic, n/N (%)	23/80 (29)	15/51 (29)	38/131 (29)	0.94^a^
Posterior rhythm, n/N (%)	18/64 (28)	22/39 (56)	40/103 (39)	0.004^a^
CSF leukocyte count, GM (GSD; min–max)	16 (6; 0–849)	20 (4; 1–259)	18 (5; 0–849)	0.46^b^
CSF antibody titer, median (IQR)	32 (16–128)	32 (10–256)	32 (16–128)	0.49^b^
Treatment				
Diagnostic delay, wk, GM (GSD; min–max)	4.0 (2.5; 0–26)	4.0 (2.6; 1–26)	4.0 (2.5; 0–26)	0.93^b^
Treatment delay, wk, GM (GSD; min–max)	3.3 (3.0; 0–24)	3.1 (2.8; 0–24)	3.3 (3.0; 0–24)	0.49^b^
First-line immunotherapy, n/N (%)	91/92 (99)	63/66 (95)	154/158 (97)	0.31^c^
IV methylprednisolone, n/N (%)	85/92 (92)	61/66 (92)	146/158 (92)	>0.99^a^
IV immunoglobulins, n/N (%)	78/92 (85)	53/65 (82)	131/157 (83)	0.59^a^
Plasmapheresis, n/N (%)	3/92 (3.3)	10/66 (15)	13/158 (8.2)	0.007^a^
Second-line immunotherapy, n/N (%)	34/92 (37)	32/66 (48)	66/158 (42)	0.15^a^
Follow-up				
Follow-up time, mo, GM (GSD; min–max)	51 (8; 2–169)	19 (11; 0–160)	36 (11; 0–169)	<0.001^b^
mRS at last follow-up, median (IQR; min–max)	1 (1–2; 0–4)	2 (1–4; 0–6)	2 (1–3; 0–6)	<0.001^b^
Good outcome (mRS ≤2) after 12 mo, n/N (%)	76/92 (89)	31/57 (54)	107/142 (75)	<0.001^a^
Returned to school/work, n/N (%)	67/92 (73)	30/65 (46)	97/157 (62)	<0.001^a^
Relapse, n/N (%)	18/92 (20)	6/67 (9)	24/159 (15)	0.065^a^

Abbreviations: GM = geometric mean; GSD = geometric SD; HSVE = herpes simplex virus encephalitis; ICU = intensive care unit; IQR = interquartile range; mRS = modified Rankin Scale.

Statistics reflect the comparison between included and excluded patients of the nationwide cohort. Radiologic and serologic findings were considered only if obtained within 90 days of disease onset, to ensure an accurate relation to disease activity. If patients had experienced a relapse in the 36 months before cognitive assessment, data from the most recent episode were included. In all other cases, clinical data are from the first or only disease episode.

a Pearson χ^2^ test.

b Wilcoxon rank sum test.

c Fisher exact test.

### Cognitive Recovery

The average timing of the neuropsychological assessments of the complete cohort was 5 years (59 months; range 0–167) after diagnosis of the encephalitis. The averaged total cognitive scores of all included participants (post-HSVE excluded) did not significantly differ from normative data (mean *z*-score −0.72 ± 0.76 SD, *p* = 0.24), as pertained to individual cognitive subdomains (Table 2).

**Table 2:** Cognitive Performance Over Time in Patients With Anti-NMDAR Encephalitis

Cognitive domain; subdomain; test	All participants (n = 74)	Months after diagnosis					
		0–6^a^ (n = 12)	6–18^b^ (n = 6)	18–36^c^ (n = 12)	36+^d^ (n = 44)		
	Mean *z*-score (SD)	Mean *z*-score (SD)	Mean *z*-score (SD)	Mean *z*-score (SD)	Mean *z*-score (SD)	n (%) <−1.5	*p* Value
Total neuropsychological assessment	−0.72 (0.76)	−1.49 (0.84)	−1.00 (0.68)	−0.69 (0.83)	−0.47 (0.57)	1 (2)^e^ 15 (34)^f^	<0.001
Memory	−0.93 (1.02)	−2.05 (0.96)	−0.95 (0.76)	−0.78 (0.97)	−0.67 (0.89)	4 (9)	<0.001
Verbal memory	−1.07 (1.15)	−2.32 (1.10)	−1.11 (0.87)	−0.96 (1.11)	−0.75 (0.99)	5 (11)	<0.001
AVLT total	−1.32 (1.22)	−2.62 (1.31)	−1.56 (0.94)	−1.25 (1.19)	−0.95 (1.00)	11 (25)	<0.001
AVLT recall	−1.14 (1.44)	−2.47 (1.56)	−1.05 (1.06)	−0.94 (1.21)	−0.85 (1.34)	7 (16)	0.004
AVLT recognition	−0.74 (1.09)	−1.87 (0.77)	−0.72 (1.04)	−0.67 (1.22)	−0.46 (0.96)	7 (16)	<0.001
Visuospatial memory	−0.81 (1.13)	−1.77 (0.90)	−0.80 (0.72)	−0.64 (1.17)	−0.58 (1.12)	10 (23)	0.011
CFT recall	−0.81 (1.13)	−1.77 (0.90)	−0.80 (0.72)	−0.64 (1.17)	−0.58 (1.12)	10 (23)	0.011
Perception and construction	−0.27 (0.88)	−1.00 (1.01)	−0.68 (0.94)	−0.18 (1.01)	−0.03 (0.68)	1 (2)	0.003
CFT copy	−0.78 (1.01)	−1.25 (0.82)	−1.11 (0.82)	−0.67 (1.07)	−0.64 (1.05)	11 (25)	0.20
JLO	0.23 (1.12)	−0.75 (1.39)	−0.24 (1.53)	0.20 (1.22)	0.57 (0.75)	2 (5)	0.001
Language	−0.97 (1.09)	−1.55 (1.07)	−1.35 (0.89)	−0.99 (1.16)	−0.75 (1.06)	12 (27)	0.11
BNT	−0.97 (1.09)	−1.55 (1.07)	−1.35 (0.89)	−0.99 (1.16)	−0.75 (1.06)	12 (27)	0.11
Attention and executive functions	−0.70 (0.84)	−1.38 (1.19)	−1.02 (0.60)	−0.82 (1.01)	−0.44 (0.56)	2 (5)	0.003
Executive functions	−0.79 (0.99)	−1.56 (1.65)	−0.92 (0.63)	−0.78 (0.89)	−0.56 (0.70)	5 (11)	0.018
Fluency animals	−1.00 (1.11)	−1.59 (1.80)	−0.97 (0.80)	−1.18 (1.04)	−0.79 (0.87)	8 (18)	0.20
Fluency occupations	−1.32 (1.08)	−2.02 (1.67)	−1.83 (1.22)	−1.50 (1.13)	−1.02 (0.69)	10 (23)	0.015
Letter fluency	−0.71 (1.08)	−1.16 (1.48)	−0.42 (0.54)	−0.62 (1.01)	−0.65 (1.02)	7 (16)	0.40
BSAT	−0.48 (1.51)	−1.59 (2.21)	−0.16 (0.87)	−0.44 (1.14)	−0.24 (1.33)	6 (14)	0.044
TMT ratio B/A	−0.42 (1.78)	−1.45 (2.96)	−1.21 (1.60)	−0.07 (1.49)	−0.12 (1.33)	7 (16)	0.074
Processing speed	−0.60 (1.10)	−1.30 (1.38)	−0.83 (0.79)	−0.67 (1.46)	−0.35 (0.87)	5 (11)	0.055
TMT A	−0.63 (1.13)	−1.11 (1.38)	−0.43 (0.70)	−0.98 (1.34)	−0.45 (1.02)	8 (18)	0.20
TMT B	−0.79 (1.51)	−1.78 (2.35)	−1.19 (1.16)	−0.79 (1.80)	−0.46 (1.04)	7 (16)	0.050
LDST	−0.36 (1.23)	−1.02 (1.15)	−0.86 (0.80)	−0.25 (1.57)	−0.15 (1.16)	5 (11)	0.12
Attention and working memory	−0.71 (0.85)	−1.26 (0.99)	−1.31 (0.52)	−0.99 (0.93)	−0.41 (0.70)	2 (5)	0.001
WAIS-IV forwards	−0.23 (1.11)	−0.45 (1.08)	−0.81 (0.85)	−0.59 (0.84)	0.01 (1.17)	2 (5)	0.14
WAIS-IV backwards	−0.79 (1.10)	−1.57 (1.25)	−1.53 (0.92)	−0.95 (1.00)	−0.45 (0.97)	6 (14)	0.004
WAIS-IV sorting	−0.61 (0.76)	−0.97 (0.73)	−0.95 (0.36)	−0.68 (0.81)	−0.46 (0.76)	3 (7)	0.13
WAIS-IV total	−1.19 (1.26)	−1.93 (1.61)	−1.94 (0.72)	−1.56 (1.39)	−0.78 (1.01)	11 (25)	0.005

Abbreviations: ALVT = Auditory Verbal Learning Test; BSAT = Brixton Spatial Anticipation Test; CFT = Complex Figure Test; HSVE = herpes simplex virus encephalitis; JLO = Judgement of Line Orientation; LDST = Letter Digit Substitution Test; TMT = Trail Making Test; WAIS = Wechsler Adult Intelligence Scale.

Cognitive performance per time of assessment in patients with anti-NMDAR encephalitis (patients with a preceding HSVE excluded). The cognitive data are reported in mean *z*-scores (SD), and overall differences over time are assessed with 1-way analysis of variance. For the long-term (>36 months) assessments, we have also included the number of patients n, (%) scoring <−1.5 SD below the norm.

a Average time of assessments 2 months ± SD 3 months.

b Average time of assessment 14 months ± SD 2 months.

c Average time of assessment 28 months ± SD 5 months.

d Average time of assessment 90 months ± SD 34 months.

e Impairment based on the total sum score.

f Impairment in at least 1 domain.

**Figure 2:**
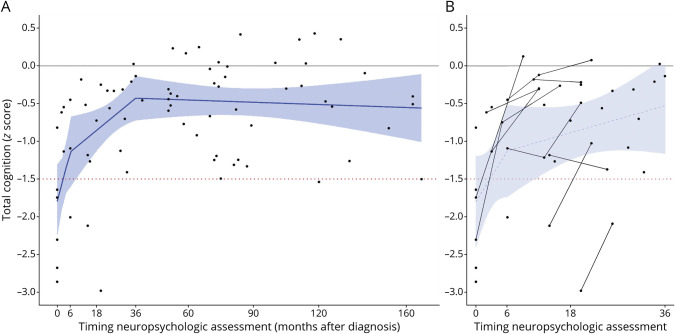
Cognitive Outcomes Improve Over Time (A) Scores on total cognition (averaged from domain-scores) are plotted in relation to normative data (*z*-score = 0 is the average score, whereas *z* = −1 represents −1 SD), against time of assessment relative to diagnosis. Every dot represents a participant (cross-sectional, patients with a preceding HSVE excluded). The blue line represents the average, depicted through linear regression with knots at the points with an evident slope change in local regression (loess). In the first 6 months after the diagnosis of anti-NMDAR encephalitis, there is a sharp increase in cognitive performance, followed by a gradual increase up to 36 months, after which it stabilizes. The red dotted line indicates the impairment level (*z*-score = −1.5 SD). (B) Scores of all cognitive assessments within the first 36 months after diagnosis (n = 30) are depicted. In this figure, follow-up assessments (n = 14) of the prospective cohort (n = 12) are included and connected with solid lines. These lines follow roughly the same temporal relation as the average of the cross-sectionally assessed patients over time (depicted as the blue dotted line). HSVE = herpes simplex virus encephalitis.

**Figure 3:**
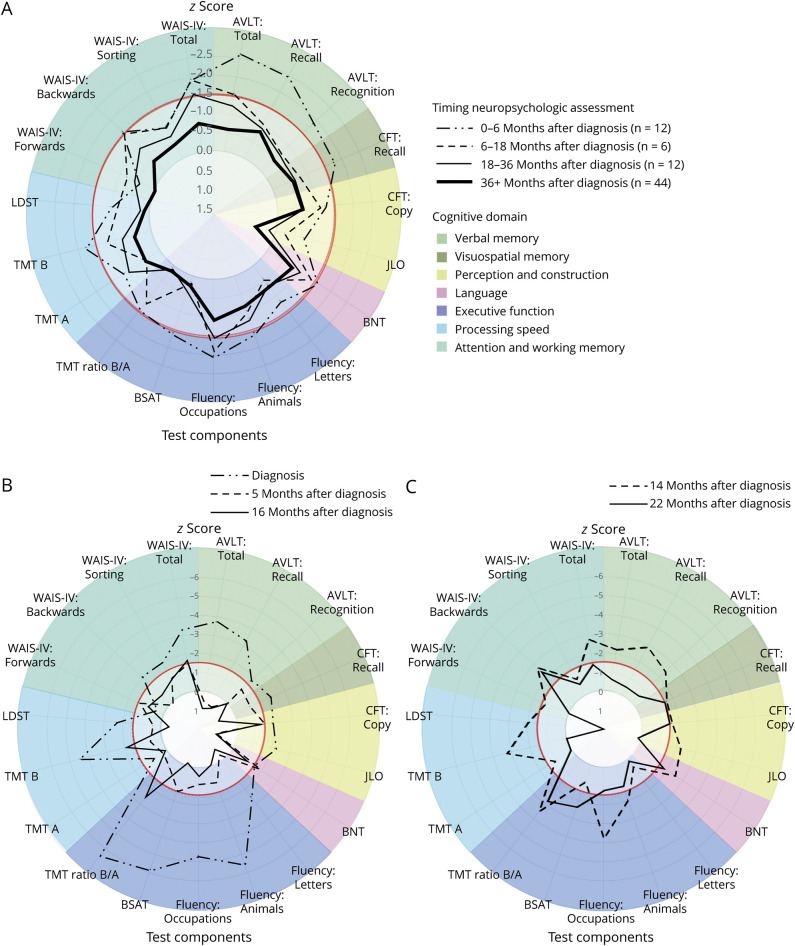
Cognitive Performance on Individual Tests and Domains Related to Passed Recovery Time After Diagnosis (A) Polar graph of the performance on individual cognitive tests, clustered per domain, of the cross-sectional cohort, split in groups based on passed time after diagnosis. The gray circle represents the average of the norm (*z*-score = 0 SD), the red dotted line the threshold for cognitive impairment (*z*-score = −1.5 SD). Individual scores are plotted about the normative data. Participants assessed after a longer recovery period after diagnosis, perform better on all tests, and regress to the norm on most cognitive domains. (B and C) Individual exemplary prospectively assessed patients. We can visualize cognitive recovery over time within patients in a similar manner. The most pronounced recovery takes place in the first months after diagnosis (B), although it can continue for years (C). ALVT = Auditory Verbal Learning Test; BSAT = Brixton Spatial Anticipation Test; CFT = Complex Figure Test; JLO = Judgement of Line Orientation; LDST = Letter Digit Substitution Test; TMT = Trail Making Test; WAIS = Wechsler Adult Intelligence Scale.

When plotting the scores against the timing of assessment after diagnosis (Figure 2), we observed an overall correlation of cognitive ability with passed recovery time since diagnosis, with a sharp increase in scores in the first 6 months, followed by a gradual increase until the 36th month (*R* = 0.35, *p* = 0.022). This temporal relation could be reproduced in the prospectively assessed patients (Figure 2B) and was consistent for all domains (Table 2, Figure 3, A–C, eFigure 2). Recently, ill participants (<6 months) had impaired (<−1.5 SD) verbal and visuospatial memory, language and executive functions, and below-average (<−1 SD) scores on processing speed, working memory and attention, and perception and construction (Table 2). Participants between 6 and 18 months after diagnosis performed better on all subdomains, with below-average scores only on verbal memory, working memory and attention, and language. Participants assessed beyond 36 months did not score below-average on any subdomain as a group, and cognitive recovery seemed to stagnate beyond 36 months (Figure 2A). However, a large variability in long-term cognitive outcomes remained; of the participants beyond 36 months postdiagnosis, 34% had an impairment (<−1.5 SD) in 1 or more cognitive domains (Table 2) and 65% scored below-average (<−1 SD). The most affected domains after 36 months were memory (mean *z*-score −0.67 ± 0.89 SD) and language (−0.75 ± 1.06).

### Post-HSVE Anti-NMDAR Encephalitis

Participants with a preceding HSVE scored significantly lower than anti-NMDAR patients without preceding HSVE on memory (mean *z*-score −2.09 ± −0.93 SD, *p* = 0.028) and language (−2.5 ± −0.97, *p* = 0.012; eTable 1).

### Patient-Reported Outcomes

**Figure 4:**
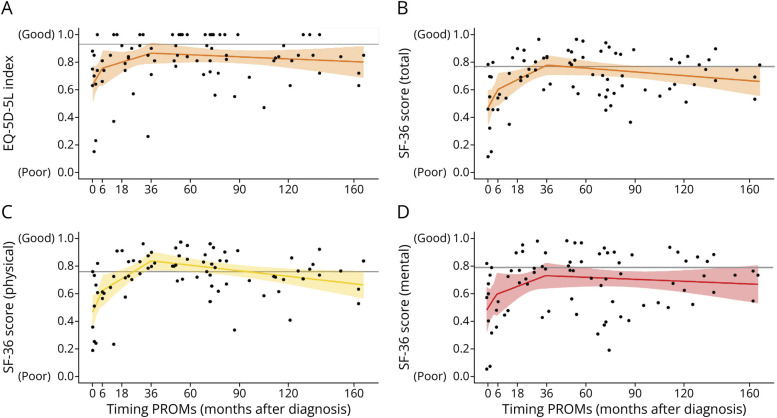
Patient-Reported Outcomes Relate to Passed Recovery Time After Diagnosis Scores on 2 exemplary self-reported outcomes (SF-36-II and EQ-5D-5L), capturing the long-term sequelae, plotted against time of assessment relative to diagnosis. Every dot represents the score of a participant (cross-sectional, patients with a preceding HSVE excluded). The colored lines represent the average, depicted through linear regression with knots at the points with an evident slope change in local regression (loess). Similar to the temporal parameters of cognitive recovery, we identify a sharp increase in scores within the first 6 months after the diagnosis of the anti-NMDAR encephalitis, followed by a gradual increase up to 36 months, after which it stabilizes. The gray horizontal line indicates the average score of the reference populations from the literature. As demonstrated in Table 2, we see self-reported physical (C) and general health (B) sequelae regress to the norm at 36 months after diagnosis, whereas self-reported mental health (D) and quality of life (A) are persistently lower compared with the reference groups, also on the long-term (>36 months). EQ-5D-5L = EuroQol 5 Dimensions 5 Levels; HSVE = herpes simplex virus encephalitis; PROM = patient-reported outcome measure; SF-36 = 36-Item Short-Form Health Survey.

A temporal relation similar to the one seen in the cognitive data was identified in the patient-reported outcomes (Figure 4, A–D, eFigure 3). Patient-reported physical problems (Figure 4C) and difficulties with daily activities (eFigure 3) in the first months after diagnosis resolved. Sequelae remained (eTable 3) in social functioning (SF-36-II; mean 73 vs norm 84, *p*-adjusted < 0.004), energy levels (SF-36-II; 57 vs 69, *p*-adjusted < 0.004), emotional well-being (SF-36-II; 72 vs 82, *p*-adjusted = 0.026, BDI-IA; 8.2 vs 5.7, *p*-adjusted = 0.010), and quality of life (EQ-5D-5L, 0.85 vs 0.93, *p*-adjusted < 0.003; Figure 4A) beyond 36 months. Self-reported sequelae in social functioning, energy levels, and quality of life were not only statistically significant but also clinically relevant, as defined in the methods.

### Prognostic Factors

Focusing on the most affected cognitive domains (memory and language) and patient-reported outcomes (SF-36-II and EQ-5D-5L), no acute factors significantly related to long-term outcome of anti-NMDAR encephalitis (eTables 4 and 5). Treatment with IV immunoglobulins showed a positive relation to quality of life (EQ-5D-5L median with IV immunoglobulin 0.90 vs 0.72, *p* = 0.02), without a relation to any other item.

### Relation of Cognitive Outcome, Patient-Reported Outcome, and Participation

In the long-term (36 months), most of the patients (91%) had a favorable outcome on the mRS (≤2)—corresponding to independence in daily activities. By contrast, 30% did not resume occupational activities (work/education), with 3% attributing this to complications unrelated to the encephalitis (this group was excluded from the analyses about occupational activities), and an additional 18% did resume occupational activities at a lower level or with adjustments relative to preillness. Patients with a relatively good cognitive outcome (total neuropsychological assessment score >−1) more often resumed work or education (82% vs 56%, *p* = 0.18), although not significantly. Participants who had resumed occupational activities scored significantly better on cognitive tests for information processing speed than participants who had not (mean *z*-score −0.14 vs −0.84, *p* = 0.039; Table 3).

**Table 3:** Relation of Long-Term Cognitive and Patient-Reported Outcomes to Resuming Work or School

Cognitive subdomains; mean *z*-score (SD)	All participants (n = 45)	Return to work/school		*p* Value	Adjusted *p* value
		Yes (n = 34)	No (n = 11)		
Total neuropsychological assessment	−0.46 (0.57)	−0.39 (0.51)	−0.69 (0.71)	0.12^a^	0.12
Verbal memory	−0.74 (1.02)	−0.60 (0.83)	−1.20 (1.43)	0.11^b^	0.11
Visuospatial memory	−0.58 (1.10)	−0.63 (1.04)	−0.41 (1.32)	0.68^c^	0.68
Perception and construction	−0.05 (0.66)	0.00 (0.66)	−0.19 (0.68)	0.22^d^	0.22
Language	−0.73 (1.07)	−0.64 (1.12)	−1.01 (0.88)	0.15^e^	0.15
Executive functions	−0.55 (0.70)	−0.45 (0.62)	−0.87 (0.87)	0.090^f^	0.090
Processing speed	−0.31 (0.89)	−0.14 (0.78)	−0.84 (1.05)	0.039^g^	0.039
Attention and working memory	−0.36 (0.72)	−0.30 (0.72)	−0.55 (0.72)	0.18^h^	0.18

PROMs (domain); median (IQR)	All participants (n = 46)	Yes (n = 35)	No (n = 11)	*p* Value	Adjusted *p* value
SF-36-II total	74.4 (60.6–84.3)	76.4 (63.9–86.3)	60.6 (52.1–79.9)	0.024^i^	0.024
Physical component	79.9 (73.6–89.1)	81.7 (74.8–91.8)	74.5 (62.3–82.0)	0.042^j^	0.084
General health	65.0 (50–80)	67.5 (51–85)	60.0 (48–65)	0.095^k^	0.19
Physical functioning	95.0 (90–100)	97.5 (90–100)	90.0 (70–95)	0.015^k^	0.060
Limitations physical health	69.0 (50–94)	72.0 (52–94)	50.0 (38–75)	0.032^k^	0.096
Pain	90.0 (68–100)	90.0 (78–100)	90.0 (68–100)	0.40^k^	0.40
Mental component	72.3 (52.3–85.5)	75.4 (54.7–86.6)	55.0 (49.5–77.3)	0.13^j^	0.13
Limitations emotional problems	75.0 (50–100)	83.0 (52–100)	58.0 (42–75)	0.061^l^	0.24
Social functioning	75.0 (50–100)	75.0 (63–100)	63.0 (50–88)	0.10^l^	0.29
Emotional well-being	75.0 (65–90)	75.0 (63–100)	70.0 (63–78)	0.095^l^	0.29
Energy	56.0 (44–69)	56.0 (44–69)	50.0 (35–69)	0.39^l^	0.39
EQ-5D-5L	0.85 (0.77–1.00)	0.90 (0.81–1.00)	0.81 (0.63–0.85)	0.008^i^	0.016

Abbreviations: EQ-5D-5L = EuroQol 5 Dimensions 5 Levels; IQR = interquartile range; PROM = patient-reported outcome measure; SF-36 = 36-Item Short Form Health Survey.

Comparisons of cognitive performance and patient-reported outcomes between participants who resumed occupational activities and participants who did not on the long-term (>36 months after diagnosis). The cognitive data and the PROM data are reported in mean (SD) and median (IQR), and differences are assessed with Welch 2 sample 1-tailed *T*-test and 1-tailed Wilcoxon rank test, respectively. *p* Values are adjusted for multiple testing with the Holm-Bonferroni method per hypothesis, according to the hypotheses that the following related to a reduced probability to resume work or education: ^a^lower overall cognitive scores relate to a reduced probability to resume work or education, ^b–h^lower scores on individual cognitive subdomains, ^i^a reduced quality of life, ^j^poorer self-reported general health relates, ^k^poorer self-reported physical health, and ^l^poorer self-reported mental health.

A relatively poor cognitive outcome (total neuropsychological assessment <−1 SD) related to a poorer self-reported mental status (SF-36-II; median 61 vs 75, *p*-adjusted = 0.030) and quality of life (SF-36-II total; 60 vs 76, *p*-adjusted = 0.034; eTable 6), although there is no strong continuous correlation (eFigure 4). Resuming work or education related to a better quality of life (SF-36-II; 76 vs 61, *p*-adjusted = 0.024 and EQ-5D-5L; 0.90 vs 0.81, *p*-adjusted = 0.016; Table 3).

## Discussion

In this study, we demonstrate that in anti-NMDAR encephalitis (1) cognitive recovery continues up to 36 months after diagnosis, with the most pronounced recovery in the initial 6 months; (2) cognitive deficits may persist years after encephalitis, most likely in the domains of memory and language, even in patients with a good outcome according to the mRS and even more pronounced in patients post-HSVE; (3) self-reported problems mostly remain in (emotional) well-being, social functioning and energy levels, most pronounced in patients with a poorer cognitive outcome; (4) nearly a third of patients has not resumed work or education after (at least) 36 months, and another 18% at a lower level or with adjustments; (5) cognitive processing speed relates to the likelihood of returning to work/education; (6) resuming work/education relates to self-reported quality of life; and (7) long-term cognitive and self-reported outcomes cannot easily be related to clinical factors in the acute phase.

In prospective and large-scale cross-sectional analyses, we demonstrate that cognitive recovery may continue for 36 months after diagnosis. Although consistent with the literature on persisting cognitive deficits years after diagnosis,^6,10,14,16,23^ a large-scale assessment of cognitive recovery over time was not available. The fast recovery in the first months after diagnosis, identified in the cross-sectional and prospective cohort, is in line with previous data prospectively collected,^34^ as is the ability to recover beyond the first year after diagnosis.^6^ The recovery trajectory in the years after diagnosis is a new insight. Although the long-term trajectory might suggest a slight decrease in cognitive functions over time, this is not a significant effect and likely confounded by the time period of diagnosis of these (cross-sectionally assessed) patients. Patients assessed 160 months (13 years) after diagnosis had been diagnosed early after the discovery of anti-NMDAR encephalitis and potentially experienced longer treatment delays and less adequate treatment—medicinal as well as rehabilitation—than the current standard.

On the long-term (>36 months postdiagnosis), over one-third of patients met the criteria of an impairment in at least 1 cognitive domain, and two-thirds scored below-average on at least 1 domain as compared with the normative population. This contrasts with a “favorable” neurologic outcome in more than 90% of the cohort captured by the mRS and aligns with previous studies noting persistent variable cognitive outcomes after recovery of other neuropsychiatric symptoms.^24^ Similarly, only 70% returned to school or work of whom a quarter returned at a lower level or required adjustments. This is slightly worse than what we reported in children before (93% resumed school, but only 64% consistently to the previous level),^16^ in line with the earlier and better recovery in children (especially adolescents) compared with older adults.^1,4^

Self-reported physical problems and difficulties with daily activities diminished over time, with remaining significant and clinically relevant sequelae in energy levels, social functioning, and (emotional) well-being on the long-term. Most self-reported sequelae were captured by the SF-36-II; self-reported implications for emotional well-being were additionally confirmed with the BDI. A diminished emotional well-being to the level of a depression has previously been demonstrated after anti-NMDAR encephalitis.^34,41^ The predisposition for emotional and social sequelae, rather than physical aspects, is in line with the literature.^23,29,30^ This conflicts with the widely accepted high recovery rates of anti-NMDAR encephalitis on the mRS because the emphasis of the mRS is more on physical independence and independence in daily activities.^41^

We identified a modest relation between self-reported mental health and quality of life with cognitive outcomes. Although we cannot determine causation, it is known that patients with a diminished emotional well-being may score worse on cognitive tests.^e15^ Alternatively, diminished well-being may result from (subtle) cognitive deficits in patients with anti-NMDAR encephalitis. The modest size of the correlation suggests that cognitive sequelae are not solely responsible for a diminished quality of life or vice versa. Factors such as coping style may play a role and should be included in future studies. The same principle holds for the relation between returning to work or education and quality of life; and whether a reduction in quality of life is a consequence of the inability to resume work or education. Causation cannot be determined; however, the 2 seem intertwined.

Verbal memory and language were the most persistent cognitive deficits. The prominent role of memory deficits in long-term outcome is in line with previous literature^6,9-18^ and with expectation based on the pathophysiology of the disease.^48^ The memory deficits correspond to structural hippocampal damage observed in imaging studies.^9^ In contrast with the literature^6,8,16,42^ and what we might expect based on the pathophysiology of the disease, executive functioning was not the second most affected cognitive domain on the long-term. This discrepancy may be attributed to the inclusion of different measures of executive functioning.^6,23^ Vice versa, the more manifested language deficits in our study were identified with a naming task. Naming tasks tap into word finding, which is closely related to (semantic) memory. Although the tasks are considered language assessments, our findings may reflect deficits in verbal memory rather than language apprehension or word formation. Deficits in executive functions, specifically processing speed, were however manifested in participants who did not resume work or education and proved important for the likelihood to reintegrate. Verbal memory, language, and executive functioning were also affected in patients not resuming work/education (eTable 7) but did not reach statistical significance, probably due to sample size.

Cognitive and patient-reported outcomes did not relate to clinical parameters in the acute phase, contrary to indications in previous research that delayed diagnosis and treatment or higher disease severity could relate to poorer cognitive outcomes.^6,33^ Clinical, radiologic, and serologic data in our study have been collected as part of standard clinical care; protocolled prospective data collection might increase the power to detect prognostic factors. Although age has previously been identified as an important predictor of long-term outcome on the mRS, we did not establish a relation with long-term cognitive outcome. A potential explanation may be that cognitive outcomes in our study were standardized accounting for age and the effect of age on cognition in the normative population, whereas the mRS does not account for age, although it is largely directed at physical aspects expected to diminish with age. The inability to predict long-term outcomes may also be attributed to the selection bias toward less severely affected patients, although this was inevitable in our cohort due to the very young age or death of a part of these patients, and is therefore likely the same in other studies. The discrepancy with other studies might therefore relate to the extended interval between diagnosis and cognitive assessment in our study. We have consciously decided to only include cognitive assessments from after the expected recovery period (36 months) in the analysis of prognostic factors, to control for confounding by the timing of the assessment. This delayed assessment has diminished the sample size (and thereby power of the study) and may have allowed for cognitive outcomes to regress toward the norm, although it may have been at a different pace. Analyzing these types of data allowing for a time-dependent outcome and combining cohorts from different studies to increase sample size may be a way to increase statistical power to identify prognostic factors (i.e., treatment delay).

The reported prevalence of persisting cognitive deficits after anti-NMDAR encephalitis, and effect on social functioning and emotional well-being, call for (1) the application of nuanced long-term outcome assessments, despite reported favorable functional outcomes and (2) extended and more comprehensive rehabilitation plans for patients recovering from anti-NMDAR encephalitis.^30^

Capturing subtle cognitive, social, or emotional sequelae of anti-NMDAR encephalitis, beyond the sensitivity of the mRS, is imperative, especially because it typically concerns young adults for whom the consequences may have large implications for participation and success in occupational, academic, and social spheres, and consequentially quality of life.^31,32^ The Clinical Assessment Scale for Autoimmune Encephalitis (CASE) has been introduced to address part of the symptoms not captured by the mRS,^49^ although it is focused on acute symptoms of encephalitis and is not as sensitive to long-term cognitive and behavioral deficits.^50^ Standardized cognitive testing and the incorporation of self-reported social abilities, participation and well-being is advised in the clinical setting, as well as in scientific research to improve the sensitivity of (long-term) outcome assessments and consequentially potentially statistical power.

In the clinical setting, the identified temporal parameters of (cognitive) recovery should be taken into consideration in rehabilitation plans, as discussed previously.^34^ Cognitive recovery takes longer than previously supposed. This challenges the effectiveness of current, often short-term, rehabilitation methods. Patients should also be counseled on the extensive period during which recovery might continue, after the initial fast(er) improvement. In addition to cognitive aspects, we emphasize the relevance of considering the personal situation, fatigability, social skills, and emotional well-being of patients in the rehabilitation process, especially because these factors seem to intertwine with cognitive recovery and reintegration in society. Patients and their social network should be informed on these potential consequences, and long-term psychotherapeutic interventions and psychosocial support should be considered as integral parts of an extended rehabilitation plan.

In spite of attempts to minimize selection bias by travelling to patients who did not have the possibilities to travel to the research institute, the included cohort is a selection of patients with anti-NMDAR encephalitis, tending toward the less severely affected in the acute phase. This may be explained by the fact that we had to exclude deceased patients and children, who were more severely affected (respectively, 76% and 60% required ICU admission, vs 43% of the nationwide cohort and 32% of included patients). Moreover, the excluded children more often had a preceding HSVE (40%, vs 13% of the nationwide cohort), and it is known that patients with a preceding HSVE are often more severely affected. Although the exclusion of these patients caused a selection bias, it was unpreventable to exclude deceased patients, and young children should be carefully assessed with tests adjusted for their age, which cannot easily be compared with the tests in this study. Patients, who were initially too severely affected to participate in the study due to a decreased consciousness, were always included in a later stage. Although this last scenario did not attribute to a selection bias of the complete included cohort, it limited the availability of results of prospectively included patients (n = 12), specifically assessments of the acute phase. Whereas the identified selection bias commands caution in generalizing results, the identified long-term effects can only be expected to be an underestimation and the implications only emphasized.

We have composed a comprehensive cognitive testing battery for this study, including psychometrically robust tests addressing the major cognitive domains in a relatively short time as to limit the burden of testing for patients.

As mentioned, conducting cognitive assessments in the acute phase of the anti-NMDAR encephalitis proved challenging due to severe symptoms or the effects of medication. Although pertinent for capturing subtle long-term effects, this implies the limited applicability of cognitive testing in the acute setting, defining a *floor effect*. It can therefore not yet replace the use of mRS or CASE scores in the acute settings of anti-NMDAR encephalitis.

Another limitation of cognitive and patient-reported data is the dependency on available normative data. Although for most cognitive tests, data of a large and diverse reference group were available, this was not always the case for patient-reported outcome measures, and reference cohorts from literature did not always reflect the anti-NMDAR encephalitis population. A higher average age of some reference cohorts (i.e., WHO-DAS-II) may lead to a relative underestimation of patient-reported sequelae.

This study delineates the temporal parameters of cognitive and psychosocial recovery after anti-NMDAR encephalitis, demonstrating early substantial gains and a prolonged recovery phase which may past 3 years. Cognitive deficits may persist at the long-term, particularly in memory and language abilities, with a third of patients performing at the level of an impairment in one of the 4 major cognitive domains. Self-reported problems most often persist in (emotional) well-being, social functioning, and energy levels. The identified cognitive and self-reported sequelae of anti-NMDAR encephalitis are not fully captured by frequently applied outcome assessments such as the mRS.

Nearly a third of patients does not resume work or education on the long-term, which relates to cognitive deficits and to self-reported well-being.

There is a need for standardized, sensitive outcome assessments in anti-NMDAR encephalitis and for extended and comprehensive rehabilitation.

## Appendix Authors

**Table:**

Name	Location	Contribution
Juliette Brenner, MD	Department of Neurology, Erasmus University Medical Center, Rotterdam, the Netherlands	Drafting/revision of the manuscript for content, including medical writing for content; major role in the acquisition of data; study concept or design; analysis or interpretation of data
Cinthia J. Ruhe, BSc	Department of Neurology, Erasmus University Medical Center, Rotterdam, the Netherlands	Drafting/revision of the manuscript for content, including medical writing for content; major role in the acquisition of data; study concept or design; analysis or interpretation of data
Ilse Kulderij, BSc	Department of Neurology, Erasmus University Medical Center, Rotterdam, the Netherlands	Major role in the acquisition of data
Anna E.M. Bastiaansen, MD, PhD	Department of Neurology, Erasmus University Medical Center, Rotterdam, the Netherlands	Drafting/revision of the manuscript for content, including medical writing for content; major role in the acquisition of data
Yvette S. Crijnen, MD	Department of Neurology, Erasmus University Medical Center, Rotterdam, the Netherlands	Drafting/revision of the manuscript for content, including medical writing for content
Chelsey N. Kret, BSc	Department of Neurology, Erasmus University Medical Center, Rotterdam, the Netherlands	Major role in the acquisition of data
Julia C.P. Verkoelen, BSc	Department of Neurology, Erasmus University Medical Center, Rotterdam, the Netherlands	Major role in the acquisition of data
Anke A.G. Tolido, BSc	Department of Neurology, Erasmus University Medical Center, Rotterdam, the Netherlands	Major role in the acquisition of data
Brigit Thomassen, MSc	Department of Neurology, Erasmus University Medical Center, Rotterdam, the Netherlands	Major role in the acquisition of data
Laura P. Kersten, MSc	Department of Neurology, Erasmus University Medical Center, Rotterdam, the Netherlands	Major role in the acquisition of data
Marienke A.A.M. de Bruijn, MD, PhD	Department of Neurology, Erasmus University Medical Center, Rotterdam, the Netherlands	Drafting/revision of the manuscript for content, including medical writing for content; major role in the acquisition of data
Sammy H.C. Olijslagers, MD	Department of Neurology, Amsterdam University Medical Center, the Netherlands	Drafting/revision of the manuscript for content, including medical writing for content
Melissa R. Mandarakas, PhD	Department of Neurology, Erasmus University Medical Center, Rotterdam, the Netherlands	Drafting/revision of the manuscript for content, including medical writing for content
Jeroen Kerstens, MD	Department of Neurology, Erasmus University Medical Center, Rotterdam, the Netherlands	Drafting/revision of the manuscript for content, including medical writing for content
Robin W. van Steenhoven, MD	Department of Neurology, Erasmus University Medical Center, Rotterdam, the Netherlands	Drafting/revision of the manuscript for content, including medical writing for content
Juna M. de Vries, MD, PhD	Department of Neurology, Erasmus University Medical Center, Rotterdam, the Netherlands	Drafting/revision of the manuscript for content, including medical writing for content
Sharon Veenbergen, PhD	Department of Immunology, Erasmus University Medical Center, Rotterdam, the Netherlands	Major role in the acquisition of data
Marco W.J. Schreurs, PhD	Laboratory of Medical Microbiology and Immunology Microvida, Tilburg, the Netherlands	Major role in the acquisition of data
Rinze F. Neuteboom, MD, PhD	Department of Neurology, Erasmus University Medical Center, Rotterdam, the Netherlands	Major role in the acquisition of data
Peter A.E. Sillevis Smitt, MD, PhD	Department of Neurology, Erasmus University Medical Center, Rotterdam, the Netherlands	Drafting/revision of the manuscript for content, including medical writing for content
Esther van den Berg, PhD	Department of Neurology & Alzheimer Center, Erasmus University Medical Center, Rotterdam, the Netherlands	Drafting/revision of the manuscript for content, including medical writing for content; study concept or design
Maarten J. Titulaer, MD, PhD	Department of Neurology, Erasmus University Medical Center, Rotterdam, the Netherlands	Drafting/revision of the manuscript for content, including medical writing for content; study concept or design

## Glossary

BDI: Beck Depression Inventory
CAE: Clinical Assessment Scale for Autoimmune Encephalitis
Erasmus MC: Erasmus University Medical Center
FSS: Fatigue Severity Scale
HADS: Hospital Anxiety and Depression Scale
HSVE: herpes simplex virus encephalitis
ICU: intensive care unit
mRS: modified Rankin Scale
NMDAR: NMDA receptor
PROM: patient-reported outcome measure
ROCFT: Rey-Osterrieth Complex Figure Test
SF-36-II: 36-Item Short-Form Health Survey II
TMT: Trail Making Test
WHO-5: World Health Organization-5 Well-being Index
WHO-DAS-II: World Health Organization Disability Assessment Schedule
